# Women, Reproductive Rights, and HIV/AIDS: Issues on Which Research and Interventions are Still Needed

**Published:** 2006-12

**Authors:** Maria de Bruyn

**Affiliations:** Policy Division, Ipas, PO Box 5027 Chapel Hill, NC 27514, USA

**Keywords:** HIV, Acquired immunodeficiency syndrome, Women, Reproductive health, Pregnancy, Human rights, Contraception, Abortion, Abortion, Induced, Interventions, Research, Literature review

## Abstract

From 2002 to 2005, two literature reviews identified a number of reproductive-health issues that appeared to be relatively neglected in relation to HIV/AIDS: contraceptive information tailored to the needs of HIV-positive people; voluntary HIV counselling and testing during antenatal care, labour, and delivery; parenting options for HIV-positive people besides pregnancy through unprotected intercourse (i.e. assisted conception and legal adoption or foster care); unwanted pregnancy; and abortion-related care. An additional finding was that stigma and discrimination were frequently cited as barriers to enjoyment of reproductive rights by HIV-positive women. Subsequently, a pilot project was initiated in which non-governmental organizations (NGOs) in developing countries used benchmarks to ascertain whether these neglected issues were addressed in local programmes and interventions serving women affected by HIV and AIDS. The benchmarks also assessed whether policies and programmes paid attention to the human and reproductive rights of HIV-positive women. This paper describes the main findings from the two exercises in relation to contraception for women living with HIV or AIDS, abortion-related care, legal adoption by HIV-positive parents, and reproductive rights. It concludes with a number of recommendations on topics to be incorporated into the international research agenda, policies, and programmes in the field of HIV/AIDS.

## INTRODUCTION

United Nations (UN) agencies now speak about feminization of the HIV/AIDS pandemic because women comprise an increasing proportion of people affected by HIV and AIDS around the world (1). They include girls, women of reproductive age, and post-menopausal women, although most new infections occur in women of childbearing age. By December 2005, it was estimated that 40.3 million people were living with HIV worldwide; of these, 17.5 million were women. The percentages of women among HIV-positive adults aged 15–49 years range from 18% in East Asia to 57% in sub-Saharan Africa; in the latter region, young women aged 15–24 years are at least three times more likely to be HIV-positive than their male peers (2).

A number of factors make women more vulnerable to HIV infection. As is well-known, women are physiologically more susceptible to infection through acts of unprotected sex than men (1). In most societies, large numbers of women are unable to insist on monogamy or consistent condom use by their male partners due to gender-based imbalances in decision-making between the sexes. Where homosexuality is heavily stigmatized, considerable numbers of men who have sex with men and who marry women to conform to societal expectations do not reveal their homosexual activity to their female partners. This may be because they fear discrimination and violence if their homosexual encounters become known or because they feel that it is none of women's business. In either case, they often do not broach use of condoms with their wives because it could cause suspicion or would prevent them from fathering children (3). Women who engage in sex work due to poverty frequently feel unable to demand use of condoms by clients. Marginalized women, such as female injecting drug users, girls, and women living on the streets who engage in transactional sex, are also often in this position.

Women are much more frequently victimized than men in domestic violence and sexual assaults in all countries of the world (4), placing them at direct risk of HIV infection and unwanted pregnancies as a result of rape. Actual and threatened psychological and physical violence also plays a role in their being unable to use contraceptives, including condoms. Poverty and lack of property rights can prevent women from leaving marriages characterized by domestic and sexual violence (5–6).

These situations, which increase the vulnerability of girls and women to HIV and sexually transmitted infections (STIs), violence, and unwanted pregnancies, clearly indicate that high priority must be given to meeting the reproductive-health needs of women. This is particularly the case for women living with HIV since their problems may be exacerbated. For example, it appears that women who disclose their HIV status may risk violence from their partners, families, or social environment (7).

To assess whether attention is indeed being paid to the broader reproductive-health needs of women affected by HIV and AIDS, literature reviews were carried out to identify which reproductive-health issues appear to be relatively neglected in relation to HIV/AIDS. The reviews showed that these issues included: contraceptive information tailored to the needs of HIV-positive people; voluntary HIV counselling and testing during antenatal care, labour, and delivery; parenting options for HIV-positive people besides pregnancy through unprotected intercourse (i.e. assisted conception and legal adoption or foster care); unwanted pregnancy; and abortion-related care. An additional finding that emerged from the reviews was that stigma and discrimination were frequently cited as barriers to enjoyment of reproductive rights by HIV-positive women.

Subsequently, a pilot project was initiated in which non-governmental organizations (NGOs) in developing countries used benchmarks to ascertain whether a number of the neglected issues identified in the literature reviews were addressed in local programmes and interventions serving affected women. The benchmarks also included attention paid to the human and reproductive rights of HIV-positive women.

This paper describes main findings of the two exercises in relation to contraception for women living with HIV and AIDS, unwanted pregnancy and abortion-related care, legal adoption by HIV-positive parents, and reproductive rights. It concludes with a number of recommendations on topics to be incorporated into the international research agenda, policies, and programmes in the field of HIV/AIDS.

## MATERIALS AND METHODS

### Literature reviews

In 2002, an initial literature review focused on factors that may influence decisions of HIV-positive women about childbearing, pregnancy outcomes in relation to HIV/AIDS, prevention of perinatal HIV transmission (PPT), and pregnancy termination by women living with HIV (8). An updated review, completed in January 2005, added some topics that seemed to be largely absent in the literature read for the first review: provision of contraceptive information tailored to the needs of HIV-positive women, critical and sensitive aspects of HIV counselling and testing during antenatal care and labour before childbirth, options for parenting other than pregnancy through unprotected intercourse, and abortion-related care (9).

The literature reviews covered more than 350 documents produced mostly after 1997. Searches were done in the CINAHL, MEDLINE, POPLINE, SOCIOFILE, and PSYCHINFO databases (key words: HIV, unwanted pregnancy, miscarriage, and abortion) and through Internet search engines (key words: contraception, contraceptives, pregnancy, PMCT or PMTCT, perinatal transmission, miscarriage, abortion, adoption, and foster care). Abstracts and materials collected at the XIII, XIV and XV International Conferences on AIDS were further included.

The documents read included research reports, scientific journal articles, conference abstracts and posters, policy and training manuals and guidelines, course materials, informational and educational materials, newspaper articles, and communications disseminated through e-mail forums. The studies were not assessed regarding the methodologies used or their research strengths and weaknesses; the aim was simply to determine whether they addressed the topics chosen and, if so, what findings or recommendations they presented.

### Pilot project in developing countries

In 2004, as follow-up to the literature reviews, Ipas collaborated with the International Community of Women Living with HIV/AIDS (ICW), the Center for Health and Gender Equity, and the Pacific Institute for Women's Health to publish a simple monitoring tool that NGOs and community-based organizations (CBOs) could use for assessing whether the reproductive-health needs of women affected by HIV and AIDS were being addressed locally (10). An underlying assumption was that many NGOs and CBOs are unable to conduct large-scale baseline and follow-up surveys and are, therefore, not in a position to measure percentage increases and decreases in various indicators. However, many organizations can collect information useful for assessing whether some progress has been made in fulfilling complete reproductive rights for women living with HIV.

The monitoring tool included a set of 17 benchmarks with accompanying questions relating to voluntary HIV counselling and testing, sexual assault, contraception, termination of pregnancy, assisted reproduction, foster care, adoption, and antiretroviral therapy and fertility issues. For example, one benchmark was: “All organizations serving HIV-positive women address all available legal options for preventing and avoiding unwanted pregnancy.” The accompanying questions to orient data collection were: How many organizations have created materials specifically for women living with HIV that discuss options for avoiding unwanted pregnancies, such as female and male condoms, microbicide research, emergency contraception, and safe legal abortion? What kinds of materials are available?

Data to answer the questions could be gathered through both qualitative (in-depth key-informant interviews, focus groups) and quantitative means (surveys). Some benchmarks could be assessed through facility visits or reviewing available documents issued by relevant organizations; for example, a review of policy statements could indicate whether agencies serving HIV-positive women publicly endorse documents listing their reproductive rights.

In 2004–2005, Ipas partnered with six civil society organizations to collect field data in seven countries using the monitoring tool (11). These partner organizations, which each produced their own country reports, were: The Federation for Women and Family Planning in Poland, the Foundation for Studies and Research on Women (FEIM) in Argentina, the Gender AIDS Forum (GAF) in South Africa, ICW in Lesotho and Swaziland, Punto de Encuentro de la Comunidad, AC (PECAC) in Mexico, and Women Fighting AIDS in Kenya (WOFAK).

As the overall project coordinator, Ipas provided sample consent forms, sample questions for interviews, and feedback on focus-group guides developed by partners. FEIM, GAF, and ICW adapted the questions slightly for their respondents. GAF, ICW, and WOFAK also organized focus-group discussions with respondents. All the partners reviewed documentation on HIV/AIDS-related policies, laws, regulations, and programmes within their countries and a selection of IEC materials. Ipas collated information from reports by the partners to produce an overall report comparing findings.

Respondents of ICW in Lesotho and Swaziland included HIV-positive women from urban and rural areas throughout those countries. The other NGOs assessed services on a more local level, including both women living with HIV and representatives of state and civil society organizations involved in HIV/AIDS-related work. In Poland, the Federation interviewed key-informants in the cities of Szczecin and Warsaw. FEIM conducted interviews in the Argentine capital of Buenos Aires and a community in Buenos Aires province. WOFAK recruited respondents in the Kenyan cities of Kisumu, Mombasa, and Nairobi. GAF carried out its research in three areas of KwaZulu Natal province close to the cities of Durban and Pietermaritzburg. Finally, PECAC interviewed key-informants in Chetumal, the capital of the Mexican state of Quintana Roo, and in Orthón P. Blanco, a larger community in that state.

## RESULTS

### Literature reviews

#### Contraception for HIV-positive women

The literature showed that agencies, such as World Health Organization (WHO), United Nations Joint Programme on HIV/AIDS (UNAIDS), and United States Agency for International Development (USAID) agree that preventing unintended pregnancies among HIV-positive women is a key way to prevent transmission of HIV to babies (10, 12–14). Nevertheless, the reviewed family-planning materials and counselling guidelines produced for developing countries not uncommonly failed to address HIV in detail.

WHO states that most contraceptive methods are appropriate for HIV-positive women based on current research evidence (15), but some considerations concerning different methods should be addressed in relation to HIV/AIDS. For example, women need to be informed that use of condoms together with another modern contraceptive can lower their risks of unintended pregnancies since rates of accidental pregnancy are higher with male and female condoms than with methods, such as pills, injectables, and implants (15). WHO has advised against generally recommending use of diaphragms and cervical caps by HIV-positive women, unless other more appropriate contraceptive methods are unavailable or unacceptable to women and further advises against initiating use of an intrauterine device (IUD) in women suffering from purulent cervicitis, chlamydial infection, or gonorrhoea (15). WHO also says that initiation of IUD use should generally not be considered for women with AIDS, unless they are clinically well on antiretroviral therapy, because potential risks usually outweigh the advantages of using the method (15).

Some drugs used to treat opportunistic infections, such as tuberculosis, may reduce the effectiveness of some oral contraceptives (16). As more women gain access to antiretroviral therapy, they also need to be informed that some antiretroviral medications may decrease the effectiveness of oral contraceptives, while oral contraceptives may increase or decrease concentrations of antiretroviral drugs (17–19). WHO recommends that women on antiretroviral therapy who use hormonal contraceptives also use condoms (15, 20), while the U.S. Department of Health and Human Services recommends that women taking certain antiretroviral drugs consider an alternative or additional method to oral contraception (17).

The literature showed that a few NGOs had published information materials that address interactions of such drugs (21–24). However, because one of their primary goals is to prevent transmission of HIV, various AIDS programmes mainly promoted use of male condoms and paid little attention to women-controlled methods to prevent pregnancy (25–28). Such observations have led to increasing calls to better integrate family-planning and HIV/AIDS programmes (11, 29, 30), although it has also been pointed out that research is needed on how such integration can best be achieved (31). A policy brief published in September 2005 pointed out that HIV-positive women still face many barriers in accessing a wide range of contraceptive services and options appropriate to their needs (32).

Given the afore-mentioned barriers and the existence of contraceptive failures, it is important that HIV-positive women have access to emergency contraception. Emergency contraception is available in many countries, but reports show that familiarity of women with, and access to, emergency contraception varies widely and can be quite low in countries as diverse as Canada, Russia, and South Africa (33–35). In Jamaica, pharmacists asked the Ministry of Health to reconsider over-the-counter availability of emergency contraception because they believe that it is over-used and led to declining sales of condoms (36).

#### Abortion-related care

While some studies on HIV and pregnancy in the literature reviews reported on pregnancy-related complications and percentages of women suffering miscarriages and stillbirths, almost none had specifically investigated induced abortion among HIV-positive women. Some researchers do not distinguish spontaneous from induced abortions when discussing pregnancy outcomes. In countries with restrictive abortion laws, hospitals may fail to record induced abortions (37) so that retrospective studies using hospital charts may have inadequate data. Data available on miscarriages and induced abortions among HIV-positive women are, therefore, likely to be incomplete.

Cases of coercion and pressure to terminate pregnancies emerged in research on other issues and also in newspaper reports (38–45). Some studies reported that women living with HIV would want to or were terminating unwanted pregnancies, even when there were numerous legal restrictions on abortion (46, 47). Reports published since the literature reviews were done have also documented such cases (48, 49).

Initiatives to better integrate family-planning and HIV/ AIDS programmes should address what HIV-positive women can do to deal with unwanted pregnancies resulting from contraceptive failure. Up to 2005, safe legal abortion was infrequently mentioned, although WHO and the international guidelines on HIV/AIDS and human rights issued by UNAIDS and the Office of the High Commissioner for Human Rights support the right of women living with HIV and AIDS to have access to safe abortion where allowed by law (50–52). A few international and national NGOs are beginning to address the rights of HIV-positive women to exercise choice in regulating their fertility (53–57), but their numbers remain limited.

#### Adoptive parenting

As access to antiretroviral therapy increases, HIV infection is becoming a more chronic rather than a fatal condition for many women and men. In this context, if cultural norms and social policies were to accept HIV-positive people as adoptive parents, some might choose to adopt rather than have their own biological children. This would require addressing cultural expectations and norms about parenting, family lineages, and inheritance that may be difficult to change. For example, focus-group participants in Malawi reported that, while childless couples might foster children, they might not receive the same respect as other parents (58). Nevertheless, work being done to alter norms about sexuality shows that such a change is possible.

Some HIV-positive women and men have indicated that they want to consider legal adoption. The topic of adoption occasionally appears on e-mail discussion groups for people living with HIV. A study among 250 HIV-positive men in São Paulo, Brazil, found that, while 52% wanted no (more) children and 5% were unsure, 43% did want (more) children in the future; 4% said that they would like to adopt a child (59).

The International guidelines on HIV/AIDS and human rights state that: “The HIV status of a parent or child should not be treated any differently from any other analogous medical condition in making decisions regarding custody, fostering or adoption” (52). Yet, the option of adoptive care may not be open to people living with HIV because of national or local policies and regulations (60, 61). Several U.S. community-based AIDS service providers interviewed in 2002–2003 reported that some of their HIV-positive clients were prohibited from visiting their children, lost custody of their children, or were not allowed to provide foster care or adopt children; this led the American Civil Liberties Union to express an interest in taking their cases to court (62). A few NGOs, such as YRG Care in India, offered their HIV-positive clients the option of adoption (63), but the topic was (and still is) scarcely addressed in the literature and in information materials for HIV-positive people.

#### Stigma and discrimination in the healthcare sector

The literature reviews revealed both anecdotal and more systematic documentation of stigmatization and discrimination against HIV-positive women in the health sector. Research in countries as varied as Burkina Faso, Burundi, the Dominican Republic, India, Indonesia, the Philippines, Russia, Uganda, the Ukraine, and Thailand showed that women often fear and actually experience HIV/AIDS-related stigmatization and discrimination in healthcare settings (39, 40, 42, 44, 64–66). An evaluation of pilot PPT programmes in Jamaica found that one reason almost half of HIV-positive pregnant women did not receive antiretroviral therapy during delivery was because they failed to reveal their HIV-positive status to healthcare workers due to fears of stigma and discrimination (67).

### Pilot project in developing countries

#### Contraception for HIV-positive women

Comparison of findings from different countries indicated that the access of HIV-positive women to general information about family-planning and contraceptive supplies varies between urban and rural areas. Respondents generally said that family-planning associations and public reproductive-health programmes often have printed materials available; access was believed to be fairly good to at least some contraceptive methods in urban areas. Poland formed an exception to this general scenario, because contraceptives are fairly expensive and not reimbursed through health-insurance schemes. However, the range of available contraceptive options appeared to be limited in all seven countries.

None of the respondents—including health professionals—said that information materials specifically addressing contraception in the context of HIV/AIDS were available or easily accessible. The health professionals in the seven countries also did not appear to often discuss family planning with women living with HIV and AIDS. The amount of contraceptive information given to HIV-positive women depended largely on preferences and attitudes of healthcare providers: most often, emphasis continued to be placed on the use of male condoms.

Results of the field studies showed that knowledge of, and access to, emergency contraception is still limited in many places. In Argentina and Poland, there was active opposition to making emergency contraception more widely available because some religious groups mistakenly claim that it is abortifacient. In Kenya, Lesotho, South Africa, and Swaziland, bureaucratic and financial factors seemed to be impediments to increased availability. The Mexican Government had just passed federal regulations stating that emergency contraception should be available through the public-health sector but implementation of the regulations had not yet begun at the time of the study.

The field studies investigated one other aspect of contraceptive use. ICW had received reports in recent years that antiretroviral-therapy programmes may require women to use provider-defined contraceptive methods to be eligible for treatment. Women living with HIV who were on antiretroviral therapy were asked about this. Some focus-group respondents in Kenya mentioned that they were asked to use condoms. Healthcare providers in Mexico said that they also promote condom use because it can help prevent infection with new strains of HIV. It was only in Lesotho that one HIV-positive woman said that she had been asked to use either injectables or an IUD so that her healthcare providers could supervise her fertility control. Women in Poland were asked to tell their physicians when they become pregnant so that their medication regimens can be changed if needed (a few antiretroviral drugs are contraindicated for pregnant women).

#### Abortion-related care

The topic of abortion—or even post-abortion care—appeared to be avoided by many respondents. A few healthcare professionals in Mexico spoke about abortion being taboo and illegal, although it is permitted in Quintana Roo State to save a woman's life, in cases of pregnancy due to rape and in cases of foetal malformation. Respondents in Lesotho and Swaziland mentioned that some women travel to South Africa for legal abortions. The Swazi women expressed great concern about the inadequacy of emergency services for post-abortion care, especially in rural areas of the country. They were also concerned about reports of abandoned babies and infanticide by women who could not cope with children resulting from unwanted pregnancies.

Even in South Africa, which permits termination of pregnancy for various reasons, the HIV-positive respondents remarked that women are dissuaded from accessing this legal medical procedure and may suffer abuse when they are able to obtain an abortion. Women spoke of being afraid to ask for an abortion at clinics and fearing that they would receive poor-quality care: “If we do access the services we are treated poorly—no respect, healthcare workers are judgmental and often cruel….” One of the worst cases of abuse reported to the project team was of a woman living with HIV who said that she was given the foetus to take home after the procedure. The nurses told her that it was her decision, so she must deal with the foetus because they would have nightmares if they had to do it. Women further reported being told that they would be given an abortion only if they agreed to be sterilized. The issue of forced sterilization was reportedly much worse in rural areas.

#### Adoptive parenting

At least some respondents in Kenya, Lesotho, and Swaziland said that they knew of HIV-positive people who had adopted children; it was unclear whether those children were also HIV-positive. In Poland, one NGO was actively mediating such adoptions, although it seemed that only HIV-positive children were placed with HIV-positive adults. In Mexico, PECAC found that governmental regulations prohibit adoption by persons living with HIV.

#### Stigma and discrimination in the healthcare sector

While some respondents and field-study researchers knew of health facilities that provide high-quality care to HIV-positive women, all the projects reported that stigma and discrimination against women living with HIV and AIDS persist within the healthcare sector. Particularly troubling was the fact that discrimination was often reported regarding obstetric and gynaecological care. The extent to which this still takes place may be much greater than is often assumed. For example, the HIV-positive researchers in South Africa were surprised at the examples of abuse given, stating that: “At the onset of the research, we were realistic about the state of the healthcare system and based on our own experiences, and that of women and girls we knew, we were aware of the negative treatment women suffered at the hands of the healthcare worker. However, the realities of what we heard and saw were far worse than we imagined…. As researchers, women and activists, we felt sad, depressed and deeply concerned about the experiences of women living with HIV and AIDS. We found it difficult to deal with the depths of grief and pain experienced by women, their lack of knowledge and access to any rights….”

The types of discrimination reported were remarkably similar across countries and regions. HIV-positive women in Africa, Europe, and Latin America are facing denial of treatment and care, humiliating and stigmatizing attitudes, and breaches of confidentiality. This is particularly the case when they encounter health professionals who are not specialized in HIV/AIDS care. The South African team also noted new types of discrimination that are taking place. Some healthcare providers require women to participate in clinic-based antiretroviral-therapy support groups as a precondition for granting them treatment. Women who had participated in antiretroviral-treatment literacy programmes and who requested particular types of treatment reported having been insulted for knowing and understanding their needs. They observed that healthcare workers often do not have adequate information themselves and then feel threatened by a treatment-literate patient.

## DISCUSSION

Although we have passed the 20-year mark for the worldwide HIV/AIDS pandemic, it is apparent that considerable numbers of healthcare professionals still have insufficient information about HIV/AIDS and their generally low risks of occupational exposure to HIV infection. This is contributing to fear among healthcare providers, who subsequently stigmatize and discriminate against patients and clients who are living with HIV in different countries (68, 69).

In reaction to the persistence of stigma and discrimination against people living with HIV/AIDS, national and international non-governmental and multilateral organizations have begun speaking out more often about the need for a human rights-based approach in HIV/AIDS-related policies and programmes. For example, in 2003, the Pan American Health Organization examined how violations of human rights are related to stigma and discrimination against HIV-positive persons, and they recommended that the international human-rights standards and norms protecting HIV-positive people be widely disseminated (70). In August 2005, UNAIDS convened a meeting to further the development of an index to measure observance of rights of HIV-positive persons and the stigma and discrimination they face (71).

The results of the field studies indicated that, while the national government programmes and hospital regulations may endorse respect for human and reproductive rights, this does not automatically translate into observance of those rights at the service-delivery level. Compliance with rights depends on enforcement by provincial and municipal authorities, funding for programmes (which affects availability of supplies, equipment, and sufficient staff), and positive attitudes and willingness on the part of service providers.

The large majority of respondents in all countries were interested in receiving more information about sexual and reproductive rights. However, both healthcare providers and women living with HIV and AIDS did not seem to be clear on how such rights could be relevant to their work environments and daily lives. Researchers in South Africa noted that healthcare workers are regarded with great respect and expected to be responsive to community needs. When they fail to do this or when they act in ways that contradict their caring role, community members are either unable to consciously identify this as a violation of their rights or are seldom able to assert their rights (for example, through direct communication with healthcare providers or by initiating a complaints procedure).

### Recommendations for research, policies, and programmes

Based on the literature review and field-study findings, several areas can be identified that warrant further policy-oriented and operational research. These include:

***The best contraceptive options for women living with HIV and AIDS***: Further research is needed on the possible interactions between hormonal contraceptives (e.g. pills, injectables, various types of implants, and contraceptive patches) and antiretroviral drugs and drugs used for treating common opportunistic infections. As new contraceptives emerge (e.g. contraceptive sprays), their effectiveness for HIV-positive women should also be assessed.

***Family-planning and pregnancy counselling for HIV-positive women and men***: Studies should focus on the quality and comprehensiveness of counselling given to HIV-positive women who are considering their reproductive options (both before and during pregnancy), particularly regarding stigmatization of women who choose pregnancy, pressure to have an abortion or to be sterilized, and withholding of information regarding post-abortion care and legal abortions for women who would want to terminate a pregnancy.

***Experiences of HIV-positive women with abortion and post-abortion care***: Such studies can examine the prevalence of coerced abortion, denial of abortion care, and unethical preconditions for granting legal abortions. Research can also determine whether HIV-positive women suffer more frequent and more severe complications from unsafe abortions and whether protocols for different types of safe abortion (e.g. vacuum aspiration or medication abortion) should take their HIV infection into account in particular ways.

A number of policy and programmatic measures can be taken to improve the situation of women living with HIV and AIDS with respect to reproductive choice and rights.

***Production and dissemination of materials on family planning, contraception, and dealing with unwanted pregnancies in the context of HIV/AIDS***: It is advisable that women living with HIV be informed about the double protection offered by male condoms against re-infection and pregnancy. However, they also should receive information about other contraceptive options in relation to HIV/AIDS (e.g. female condoms, which contraceptives might be preferable for women in their situation, potential interactions between hormonal contraceptives and drugs for opportunistic infections and antiretroviral therapy). Options for avoiding unwanted pregnancies should be mentioned, including emergency contraception and safe abortion for indications considered lawful in each country. Given the high prevalence of sexual violence experienced by women living with HIV, for example, it is important that they know whether pregnancy termination is allowed in cases of rape or when the health of a woman is endangered. Such materials can be produced through collaboration among AIDS service organizations, reproductive health/family-planning programmes, NGOs, and associations of people living with HIV and AIDS. Work on these materials can also be combined with lobbying and advocacy work to expand options of women to exercise their rights to health and to decide whether and when to have children.

*Exploration of possibility of HIV-positive women and men adopting children*: While HIV-positive persons can reduce the chances of transmission of HIV by participating in prevention of perinatal transmission of AIDS programmes, they can also avoid perinatal transmission by avoiding pregnancy. Many HIV-positive people, particularly younger women, do want biological children. However, some are interested in adopting children. Organizations working on AIDS should ascertain whether legal restrictions on adoption prevent HIV-positive people from adopting and, if so, whether these restrictions are reasonable. NGOs and government agencies entrusted with adoption programmes should collaborate with associations of people living with HIV to discuss how programmes can ensure that HIV-positive people are not automatically disqualified as prospective adoptive parents. Adoption as a parenting option should be included in informational materials on sexual and reproductive health for people affected by HIV. Associations of people living with HIV can also begin disseminating stories about successful adoptions through newsletters, websites, and conferences; cases from one country can serve as examples and inspiration for people in other countries.

***Expanded training for health professionals on occupational risks in conjunction with capacity-building on the rights of people living with HIV and AIDS***: Such training should emphasize that it is a human right for healthcare providers to have sufficient supplies available to observe universal precautions (e.g. gloves, disposable needles, sharps disposal boxes). Health systems must also make post-exposure prophylaxis (antiretroviral drugs to prevent HIV infection) available to providers. Training on the ethics of service provision to people living with HIV should incorporate HIV-positive women as paid facilitators who can lead dialogues on how attitudes affect treatment of clients and patients. HIV-positive women who have had treatment-literacy training can help explain the importance of patients being partners with health professionals in implementation of treatment.

***Dissemination of information on human-rights standards in practical terms relevant to work and lives of HIV-positive women and care providers***: It is important to inform people living with HIV and AIDS, service and healthcare providers, NGO staff, and policy-makers about sexual and reproductive rights in the context of HIV/AIDS. A first step can involve widely disseminating documents that identify and explain sexual and reproductive rights (52, 72, 73). Summaries in the form of brochures can be distributed through NGOs, waiting rooms of governmental agencies, voluntary counselling and testing sites, hospitals, and clinics.

To help people understand how these rights apply to their lives, however, dissemination of information is not enough. They need to understand which international rights treaties their government has ratified, how those rights can be claimed through laws and regulations (which requires lobbying lawmakers), and how they can bring forward complaints when rights are violated. This can include instructions on how to lodge complaints with hospital and clinic directors or ethics committees, how to submit cases for follow-up with local human rights commissions and ombudsmen, and how to find lawyers who could take well-documented cases to court if necessary. Pressure can also be put on the government to speed up compliance with human-rights treaties by preparing shadow reports for international committees that monitor treaty compliance; such reports can come from individual citizens and NGOs.

Information about these practical measures can be disseminated through articles in newsletters and journals, through brochures and leaflets, and through capacity-building sessions for both service providers and clients/patients. When people understand that there are concrete steps they can take regarding discrimination and violations of rights, these rights can leave the realm of theory and enter into the reality of daily life.

The findings that emerged from the literature reviews and pilot project show that much remains to be done to meet the comprehensive health needs and fulfill the reproductive rights of HIV-positive women. This includes devoting more research funds and human resources to investigating issues relating to reproduction, especially topics that are largely neglected or considered sensitive or contentious, such as termination of pregnancy and adoption.

A basic premise underlying the piloted monitoring tool is that awareness of reproductive rights is essential for providing HIV-positive women with comprehensive healthcare. All countries in the United Nations have endorsed at least one international human-rights convention establishing a right to health and healthcare. Therefore, all UN Member States are obliged to respect, protect, and fulfill this right, including for women living with HIV and AIDS.

If women living with, and affected by HIV and AIDS, can become better-informed advocates for those rights, they can hold their governments and non-state actors accountable for fulfilling their rights. The pilot project presented the staff of some partner organizations with their first opportunity to engage in a simple research/data-collection exercise. Team members in Kenya said that they became more conscious of the importance of the assessed issues: “It is evident that…we need to lobby and advocate for better policies on issues pertaining to sexual [and] reproductive health.” PECAC commented that their project was the first to focus on the study topics in their locality, and they used the findings to obtain funding for concrete community action projects. Respondents also found the pilot project to be a valuable exercise; some even commented that their participation motivated them to begin researching the issues to increase their knowledge on issues they had not yet considered.

Such observations highlight the need to enable HIV-positive women to assume positions as policy-makers, programme managers, and researchers. Many women who could contribute invaluable information to inform and direct policies and programmes on reproductive rights and HIV/AIDS lack formal training on how to translate their experience into advocacy and action. If these women are to have a meaningful place at decision-making tables, they will need support to become effective advocates. NGOs and universities can provide HIV-positive women with a wealth of information on advocacy and policy-making strategies and procedures. They can further contribute to enhancing skills of women, for example, by engaging more women in gathering information and evidence on which advocacy and community action can be based—similar to what was done in the field studies reported in this paper.

## ACKNOWLEDGEMENTS

The following women collaborated in carrying out the monitoring tool project: Argentina—María Ines Ré and Mabel Bianco of FEIM; Kenya—Monique Wanjala and Dorothy Onyango of WOFAK; Lesotho and Swaziland—Luisa Orza and Emma Bell of the International Community of Women Living with HIV/AIDS (ICW); Mexico—Lorena Careaga Viliesid of Punto de Encuentro con la Comunidad AC; Poland—Anna Domaradzka and Wanda Nowicka of the Federation for Women and Family Planning; and South Africa—Lucky Barnabus, Dawn Cavanagh, Ntokozo Madlala, Thenjiwe Magwaza, Thandeka Maphumulo, Nothile Mcanyana, Rosemary Mlambo, Gugu Mpungose, Cindy Ngidi, Vicci Tallis, Rose Tolofi of the Gender AIDS Forum. The monitoring tool was revised after a second phase of the project; the updated version is available at: http://www.ipas.org/publications/en/MDGHIV_E06_en.pdf.
